# High pressure Raman spectroscopy of H_2_O-CH_3_OH mixtures

**DOI:** 10.1038/srep08532

**Published:** 2015-02-23

**Authors:** Wen-Pin Hsieh, Yu-Hsiang Chien

**Affiliations:** 1 Institute of Earth Sciences, Academia Sinica, Nankang, Taipei. 11529, Taiwan

## Abstract

Complex intra-molecular interactions and the hydrogen-bonding network in H_2_O-volatile mixtures play critical roles in many dynamics processes in physical chemistry, biology, and Earth and planetary sciences. We used high pressure Raman spectroscopy to study the pressure evolution of vibrational frequencies and bonding behavior in H_2_O-CH_3_OH mixtures. We found that the presence of low CH_3_OH content in H_2_O increases the transition pressure where water crystallizes to ice VI, but does not significantly change the pressure where ice VI transforms to ice VII. Furthermore, the stiffening rates of C-H stretching frequencies *dω*/*dP* in CH_3_OH significantly decrease upon the crystallization of water, and the softening rates of the O-H stretching frequencies of ice VII are suppressed over a narrow pressure range, after which the frequencies of these modes shift with pressure in ways similar to pure CH_3_OH and ice VII, respectively. Such complex pressure evolution of Raman frequencies along with pronounced variations in Raman intensities of CH_3_OH within the sample, and the hysteresis of the water-ice VI phase transition suggest pressure-induced segregation of low content CH_3_OH from ice VII. These findings indicate the significant influence of volatiles on the crystallization of sub-surface ocean and thermal evolution within large icy planets and satellites.

H_2_O is the archetypal hydrogen-bonded and the most abundant polyatomic molecule in the universe^1^. It displays a rich pressure-temperature phase diagram^2^, and the evolution of its molecular vibrational behavior under extreme pressures has been a major area of broad research interest^3^,^4^,^5^,^6^,^7^. In addition to pure H_2_O, knowledge of the high pressure physical and chemical properties of H_2_O mixed with other volatiles, such as methanol (CH_3_OH), methane (CH_4_), ammonia (NH_3_), and carbon dioxide (CO_2_), is critical to understanding many phenomena in Earth and planetary systems, e.g., the effect of volatiles on the geodynamics and thermal history of icy satellites^8^,^9^,^10^,^11^,^12^. Among simple volatiles, CH_3_OH is also a prototypical, polar and hydrogen-bonded molecule; when mixed with water it serves as an anti-freeze compound that lowers the freezing temperature of water at ambient pressure^11^. It has been shown that the presence of CH_3_OH in H_2_O could affect the crystallization behavior of the primordial ocean of icy satellites and determine whether a sub-surface ocean can be formed^10^.

The inter- and intra-molecular interactions and molecular structure of H_2_O-CH_3_OH mixtures with various relative concentrations have been extensively studied using computer simulations^13^,^14^,^15^,^16^,^17^,^18^,^19^, neutron and X-ray diffraction and spectroscopy techniques^20^,^21^,^22^,^23^,^24^,^25^, and Raman spectroscopy^26^,^27^,^28^,^29^. At ambient conditions, the H_2_O water is composed of a tetrahedral, hydrogen-bonded molecular structure^30^, whereas the CH_3_OH is composed of hydrogen-bonded chains or rings^20^. Upon mixing, the local arrangement and structure of these molecules deviate from those of each pure phase and could change with different relative concentrations due to the complex hydrogen-bonded, hydrophobic, and hydrophilic interactions. For instance, recent studies have shown that at ambient conditions, a mixture with higher than 50% molar fraction of CH_3_OH exhibits incomplete mixing at the molecular level, i.e., H_2_O molecules form clusters and segregate from the CH_3_OH^15^,^20^,^21^; the molecular segregation is enhanced by cooling and moderate compression due to the formation of larger water clusters^24^. However, previous studies have largely been performed near ambient conditions; the effect of extreme pressure on the local molecular bonding and structure have been rarely investigated and only up to the hundreds of MPa range, much lower than the relevant conditions in planetary and icy satellite interiors, where H_2_O is typically mixed with other volatiles (less than 20 wt%) under a few GPa pressures^9^,^10^.

Raman spectroscopy enables the probing of local vibrational modes of H_2_O-CH_3_OH mixtures and thus offers a powerful means for monitoring the evolution of both intra- and inter-molecular bonding under high pressure conditions that is complementary to X-ray and neutron diffraction techniques. In this work, we present a high pressure Raman spectroscopy study of H_2_O mixed with less than 20 wt% (<12% molar fraction) CH_3_OH at room temperature to approximately 10 GPa, a pressure range over which the liquid water undergoes pressure-induced phase transitions to crystalline ice VI and ice VII phases. Pressure tuning of the inter- and intra-molecular interactions and structures over a broad pressure range allows us to characterize the evolution of molecular vibrational frequencies and the bonding behavior of the mixtures. We observed a significant influence of CH_3_OH on the crystallization of compressed H_2_O at high pressure, i.e., the pressure where liquid H_2_O crystallizes to ice VI at room temperature increases with increasing CH_3_OH content, whereas upon decompression ice VI melts to water at nearly the same pressure as in pure H_2_O, independent of the CH_3_OH content. In addition, it is well known that pressure typically enhances the inter-molecular interactions and could create additional bonding between different species, synthesizing novel molecular compounds^31^,^32^,^33^,^34^. In our experiments we observed intricate evolution of the C-H stretching frequencies of CH_3_OH and the O-H stretching frequencies of ice VII under pressure as well as pressure-induced variations in Raman intensities of the C-H stretching modes of CH_3_OH within the sample. These findings combined with the hysteresis of the water-ice VI phase transition suggest pressure-induced segregation of low content CH_3_OH from the ice VII.

## Results

**Figure 1:**
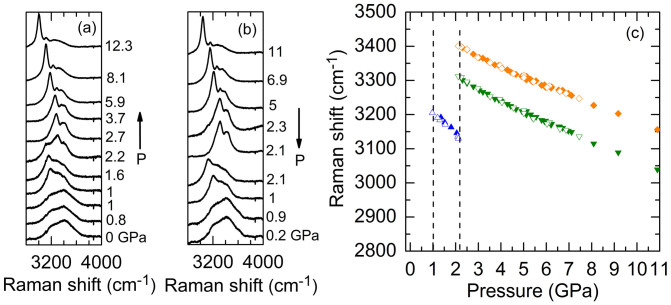
Representative pressure dependence of the Raman spectra of H_2_O during (a) compression and (b) decompression cycles. Pressure is labeled next to each spectrum which is displaced vertically for clarity. During the compression cycle, the liquid water crystallizes to ice VI at 1 GPa and ice VI transforms to ice VII at 2.2 GPa where coexistence of the two phases is observed in the spectrum. (c) Raman shifts of the O-H stretching modes of H_2_O as a function of pressure during compression (filled symbols) and decompression (open symbols) cycles. The dashed lines mark the phase transition pressures for compressed water-ice VI and ice VI–VII.

Figures 1a and 1b show representative Raman spectra of H_2_O at room temperature as a function of pressure during compression and decompression cycles. We focused on the frequency range of 2800 to 4000 cm^−1^ and observed two broad Raman bands around 3235 and 3400 cm^−1^ due to the O-H stretching modes of liquid water. Upon increasing pressure, water then crystallized to ice VI at 1 GPa accompanied by the appearance of a characteristic, intense Raman mode around 3180 cm^−1^. Ice VI further transformed to ice VII at approximately 2.2 GPa, and coexistence of the ice VI and VII was observed during a pressure range from 2.2 to 2.3 GPa (Fig. 1a). Upon decompression, the ice VII reversibly returned to ice VI at 2.1 GPa, which then transformed back to the liquid phase at 0.9 GPa. In Fig. 1c, we plot the pressure dependent Raman shifts of the O-H stretching modes of ice VI around 3200 cm^−1^ and ice VII around 3300 and 3400 cm^−1^. The vibrational frequencies of the O-H stretching modes all red-shifted with increasing pressure due to the strong hydrogen-bonded network between the H_2_O molecules. All the frequency shifts were reversible upon decompression. The pressure dependent Raman spectra, phase transition pressures between liquid and crystalline phases, and Raman shifts under pressure are well consistent with previous studies^6^,^7^,^35^.

**Figure 2:**
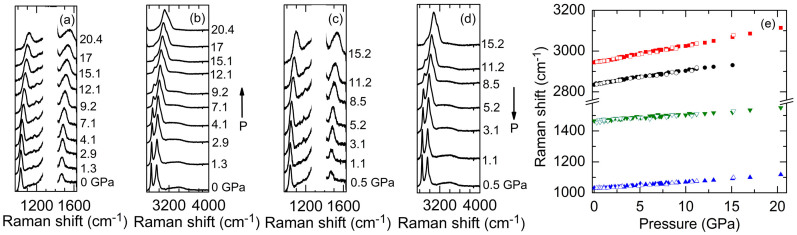
Representative pressure dependence of the Raman spectra of CH_3_OH during (a) (b) compression and (c) (d) decompression cycles. Pressure is labeled next to each spectra which are displaced vertically for clarity. (e) Raman shifts of the CH_3_OH for C-O stretching around 1030 cm^−1^ (upper triangles), C-H bending around 1465 cm^−1^ (lower triangles), and C-H stretching around 2835 cm^−1^ (circles) and 2944 cm^−1^ (squares) during compression (filled symbols) and decompression (open symbols) cycles. The frequencies of all four modes shifted approximately linearly and reversibly over the pressure range studied. In (a) and (c), the strong Raman signal from the diamond anvils around 1330 cm^−1^ is removed for clarity.

The representative pressure dependence of Raman spectra of CH_3_OH, the other end member of H_2_O-CH_3_OH mixture, is shown in Figs. 2a–d. At ambient pressure, we observed four dominant modes, including a C-O stretching mode at 1031 cm^−1^, a C-H bending mode at 1465 cm^−1^, and two C-H stretching modes at 2835 cm^−1^ (symmetric stretching) and 2944 cm^−1^ (anti-symmetric stretching) that are due to the Fermi resonance^36^. In addition, we also detected small, broad bands around 3300 and 3400 cm^−1^ due to the O-H stretching modes. All the characteristic Raman modes are in good agreement with literature results^26^,^29^,^36^,^37^,^38^. Upon compression, all the dominant modes blue-shifted and broadened with increasing pressure^38^. Moreover, the ratio of the Raman intensity of the C-H anti-symmetric stretching to symmetric stretching mode increased dramatically, resulting in a broad spectrum when the pressure is higher than 17 GPa. During the decompression cycle, a reversible evolution with pressure was observed where the C-O stretching and C-H bending modes returned to the initial spectrum before compression, and the pressure-induced broad spectrum of the C-H stretching modes transformed back to the starting two peaks with the same initial Raman intensity ratio.

We summarize the Raman shifts of the four dominant modes of CH_3_OH as a function of pressure in Fig. 2e. All the frequencies shifted linearly with pressure during both the compression (filled symbols) and decompression (open symbols) cycles. We did not observe mode splitting and discontinuous frequency shifts that are signatures of the pressure-induced crystallization of liquid CH_3_OH^37^, indicating that in our experiments the CH_3_OH was superpressed and did not transform into a crystalline phase, as reported in previous studies that under pressure CH_3_OH could be difficult to crystallize and usually forms a superpressed liquid^37^,^39^.

**Figure 3:**
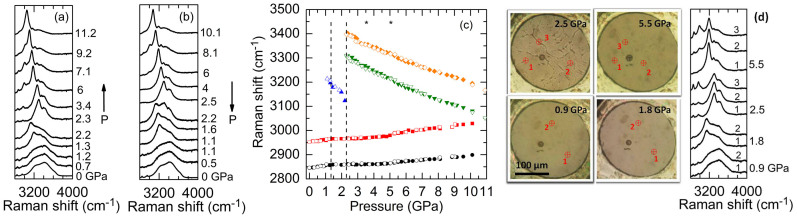
Representative pressure dependence of the Raman spectra of H_2_O mixed with 2.5 wt% CH_3_OH during (a) compression and (b) decompression cycles. Pressure is labeled next to each spectrum which is displaced vertically for clarity. With the addition of 2.5 wt% CH_3_OH, the compressed liquid water crystallizes to ice VI at 1.3 GPa and ice VI transforms to ice VII at 2.3 GPa. (c) Raman shifts of the O-H stretching modes of H_2_O and the C-H stretching modes of CH_3_OH as a function of pressure during compression (filled symbols) and decompression (open symbols) cycles. The slopes *dω*/*dP* of the C-H stretching modes decrease upon crystallization of the compressed liquid water and then increase around 5 GPa. The softening rates of the O-H stretching modes of ice VII decrease around 3.5 GPa and slightly increase after 5 GPa. The two stars indicate the pressure range over which the reduction in the softening rates of the O-H modes occurs. The dashed lines mark the phase transition pressures for compressed water-ice VI and ice VI–VII, respectively. (d) Representative photomicrographs of the 2.5 wt% CH_3_OH mixture at high pressures. The cross-circles in each photomicrograph mark the locations where the Raman spectra were taken within the DAC and their corresponding spectra numbered above each one are shown in the right panel. Significant variations in the Raman intensities of the C-H stretching modes of CH_3_OH and the O-H stretching modes of ice VII are observed at 2.5 and 5.5 GPa. Note that the Raman spectra were taken at locations similarly distant from the center of the DAC to minimize the pressure gradient among different locations. The minimal pressure difference is confirmed by the observation that the Raman shifts measured at different locations are essentially identical.

Addition of CH_3_OH to H_2_O changes the local molecular bonding in both species and therefore, the phase transition behavior under pressure. The representative pressure dependence of Raman spectra of H_2_O mixed with 2.5 wt% CH_3_OH at room temperature during compression and decompression cycles are shown in Figs. 3a and 3b. At ambient pressure we observed two strong and broad Raman bands of liquid water around 3240 and 3410 cm^−1^ as well as two small peaks at 2844 and 2952 cm^−1^ due to the C-H stretching of CH_3_OH, in good agreement with literature^26^,^40^. At 1.3 GPa the liquid water crystallized to the ice VI, which then transformed to the ice VII phase at 2.3 GPa. The higher crystallization pressure compared to pure H_2_O indicates that the CH_3_OH serves as an anti-freeze compound that stabilizes liquid water (expanding the pressure range of stability of water) and delays its crystallization under pressure, similar to its anti-freeze effect whereby it lowers the freezing temperature of liquid water at ambient pressure. During decompression, the ice VII reversibly returned to ice VI at 2.2 GPa, whereas the ice VI transformed back to the liquid water at 1.1 GPa. Since the uncertainties of the pressure measurements are typically ~0.1 GPa, the slightly lower transition pressure from ice VI to water compared to that during compression (1.3 GPa) indicates hysteresis water-ice VI transition under pressure due to the presence of CH_3_OH. This hysteresis effect is enhanced with higher CH_3_OH contents as presented below.

Figure 3c summarizes the pressure dependent Raman shifts of the O-H stretching modes of ice VI and VII as well as the C-H stretching modes of CH_3_OH for H_2_O mixed with 2.5 wt% CH_3_OH. Three experimental runs yielded consistent results. The frequencies of the two C-H stretching modes initially increased linearly with pressure, but the slopes *dω*/*dP* substantially decreased around 1.3 GPa, when the liquid water crystallized to ice VI. Similar to the pressure dependence of the Raman shifts in pure H_2_O, the frequencies of the O-H stretching modes of ice VI and VII all red-shifted with increasing pressure. The softening rates of the O-H stretching modes of ice VII, however, were suppressed during a pressure range from approximately 3.5 to 5 GPa (labeled by two stars in Fig. 3c). After 5 GPa, the softening rates increased and became similar to the softening rates of pure ice VII; meanwhile the C-H frequencies of CH_3_OH started to increase linearly with larger slopes than those during 1.3 to 5 GPa. During the decompression cycle, all the frequency shifts were reversible, except that around 1.1 GPa the O-H frequency of ice VI was still observed due to the delayed transition from ice VI to liquid water, and that when *P* < 1 GPa the small slopes for the C-H frequencies with pressure, *dω*/*dP*, increased and became similar to the slopes during initial compression.

During the compression cycle, we also observed clear changes in the distribution of the mixture within the sample chamber of the diamond anvil cell (DAC) (Fig. 3d). When the H_2_O is in the liquid phase (*P* < 1.3 GPa), the 2.5 wt% CH_3_OH mixture was transparent and the Raman spectra taken at different locations within the DAC were nearly the same, see for instance, the photomicrograph and spectra at 0.9 GPa in Fig. 3d. However, upon crystallization of water to the ice VI and VII, the Raman spectra taken at different locations within the DAC showed pronounced variations in the intensities of the C-H stretching modes of CH_3_OH and the O-H stretching modes of ice VI and VII, e.g., the spectra at 2.5 and 5.5 GPa in Fig. 3d, indicating a pressure-induced inhomogeneous distribution that large amount of CH_3_OH molecules likely aggregates at the boundaries of the ice domains. Such pressure-induced molecular segregation was also observed in all other higher CH_3_OH content mixtures as discussed below.

The transition pressure and pressure dependent Raman shifts were further investigated with H_2_O mixed with 5, 10, and 20 wt% CH_3_OH. Similar to the 2.5 wt% CH_3_OH mixture, at ambient pressure all these mixtures also showed two broad bands of liquid water around 3240 and 3410 cm^−1^ and two small peaks at 2844 and 2952 cm^−1^ thanks to the C-H stretching of CH_3_OH. During compression, the pressure where water crystallized to ice VI was further raised with higher CH_3_OH content, i.e., it increased to 1.5 GPa for 5 wt% CH_3_OH, 2 GPa for 10 wt% CH_3_OH, and 2.1 GPa for 20 wt% CH_3_OH, respectively. The ice VI–VII transition, however, all occurred around 2.3 to 2.4 GPa, the same as that in 2.5 wt% CH_3_OH mixture. When releasing pressure, the ice VII reversibly transformed back to ice VI at 2.3 GPa in all these mixtures, but ice VI underwent a hysteresis phase transition and melted to liquid water at pressures around 0.8 to 1.1 GPa, much lower than the crystallization pressure during compression.

**Figure 4:**
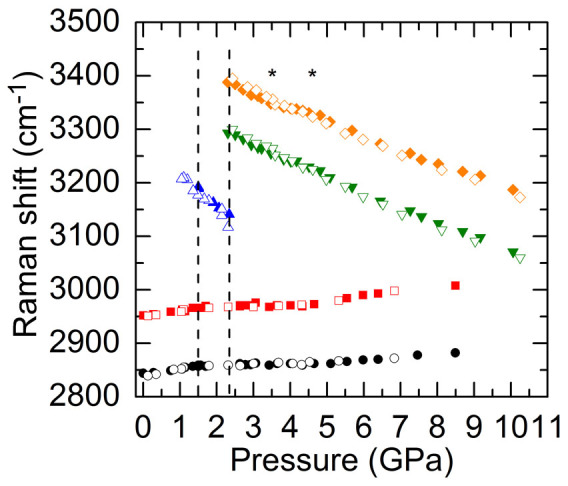
Pressure dependent Raman shifts of the O-H stretching modes of H_2_O and the C-H stretching modes of CH_3_OH during compression (filled symbols) and decompression (open symbols) cycles for H_2_O mixed with 5 wt% CH_3_OH. The stiffening rates of the C-H stretching modes decrease around 1.5 GPa when water crystallizes to ice VI, and then increase again around 4.6 GPa with larger slopes than those between 1.5 and 4.6 GPa. The softening rates of the O-H stretching modes for ice VII are reduced during a pressure range from approximately 3.4 to 4.6 GPa, which is labeled by two stars. The dashed lines mark the phase transition pressures for compressed water-ice VI and ice VI–VII, respectively.

**Figure 5:**
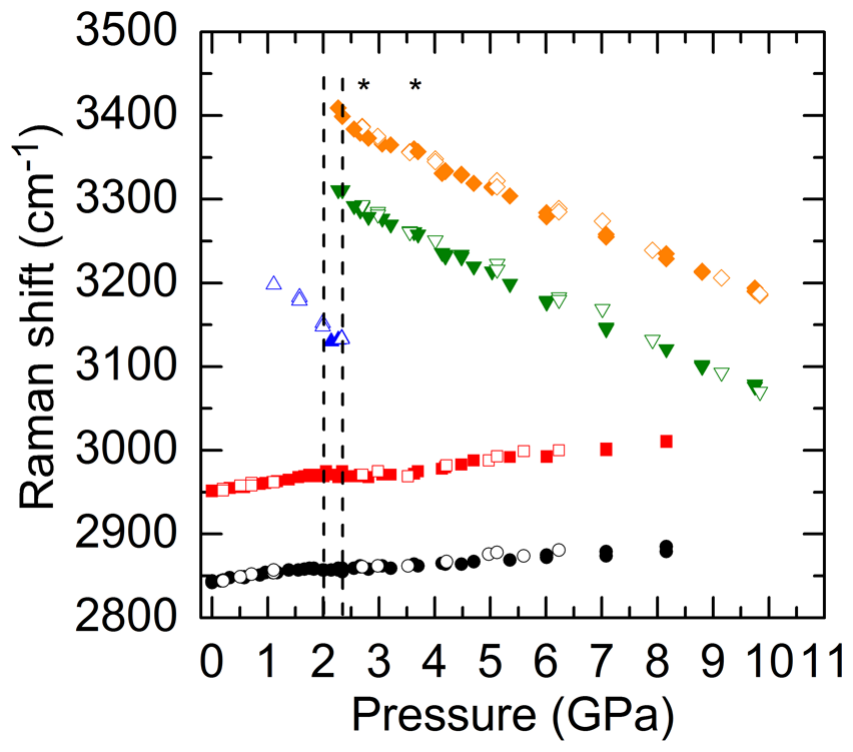
Raman shifts of the O-H stretching modes of H_2_O and the C-H stretching modes of CH_3_OH during compression (filled symbols) and decompression (open symbols) cycles for H_2_O mixed with 10 wt% CH_3_OH. The stiffening rates of the C-H stretching modes of CH_3_OH decrease upon the crystallization of water and then increase around 3.6 GPa with larger slopes. On the other hand, the softening rates of the O-H stretching modes for ice VII experience a reduction between approximately 2.7 and 3.6 GPa (labeled by two stars). The dashed lines mark the phase transition pressures for compressed water-ice VI and ice VI–VII, respectively.

**Figure 6:**
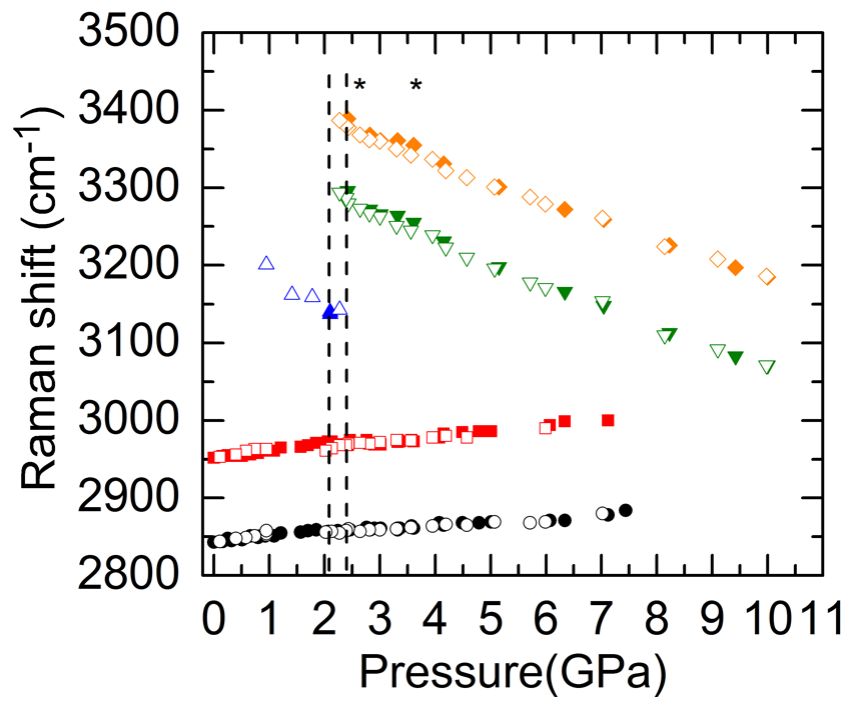
Raman shifts of the O-H stretching modes of H_2_O and the C-H stretching modes of CH_3_OH during compression (filled symbols) and decompression (open symbols) cycles for H_2_O mixed with 20 wt% CH_3_OH. The slopes *dω*/*dP* of the C-H modes decrease when the compressed water crystallizes to ice VI and then increase after 3.6 GPa. The softening rates of the O-H modes decrease between approximately 2.6 and 3.6 GPa (labeled by two stars), after which they increase and become similar to those of pure ice VII. The dashed lines mark the phase transition pressures for compressed water-ice VI and ice VI–VII, respectively.

The pressure dependent Raman shifts of the O-H stretching modes of ice VI and VII and the C-H stretching modes of CH_3_OH for H_2_O mixed with 5, 10, and 20 wt% CH_3_OH are plotted in Figs. 4, 5, and 6, respectively. Similar to the H_2_O-2.5 wt% CH_3_OH mixture, the frequencies of the two C-H stretching modes in all these mixtures initially increased linearly with pressure until the pressures where liquid water crystallized to ice VI and then the slopes *dω*/*dP* became very small. The O-H stretching modes of ice VI and VII, on the other hand, all softened with pressure. The softening rates for the ice VII were also suppressed during a narrow pressure range (labeled by two stars in Figs. 4, 5, and 6), after which they softened with larger slopes similar to the pure ice VII, and meanwhile the small slopes of the C-H stretching modes of CH_3_OH increased. Note that the suppression in the softening rates of O-H modes for ice VII in 5 wt% CH_3_OH mixture occurs at higher pressure regime compared to 10 and 20 wt% CH_3_OH mixtures. During decompression, all frequencies reversibly shifted until the pressures where compressed water crystallized to ice VI, below which the ice VI phase was still present due to the delayed ice VI-water transition; when *P* < 1 GPa the small slopes of C-H frequencies in all these mixtures increased to similar slopes upon initial compression.

**Figure 7:**
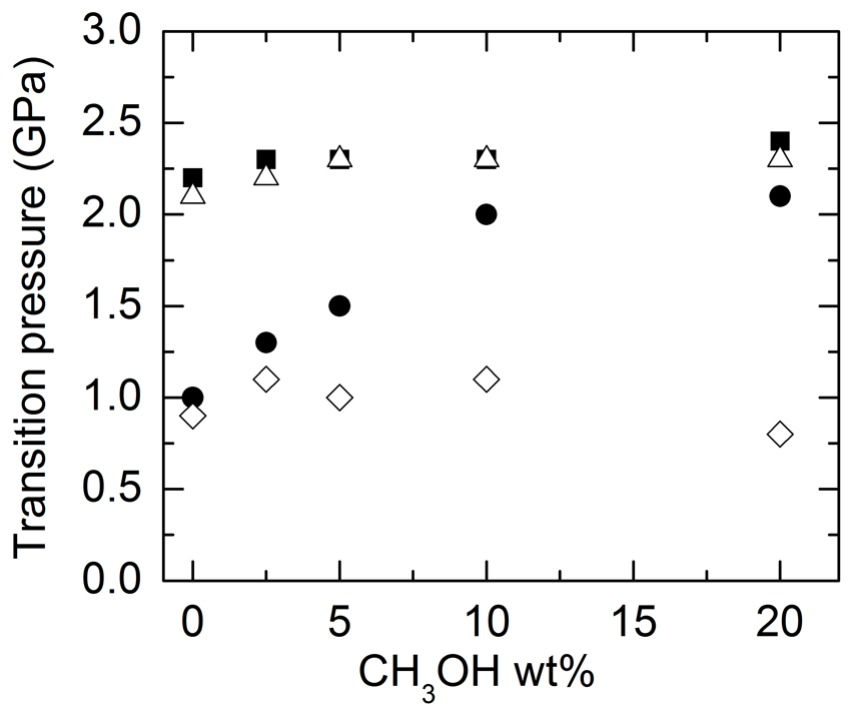
Transition pressures for water-ice VI and ice VI–VII as a function of CH_3_OH wt %. In the compression cycle, the crystallization pressure of water (filled circles) significantly increases with the CH_3_OH wt%, but the transition pressure of ice VI to VII (filled squares) is weakly dependent on the CH_3_OH wt%. During the decompression, the ice VII transforms back to the ice VI (open triangles) at approximately the same pressure as being compressed; the ice VI, however, experiences a hysteresis and melts to water around 0.9 GPa (open diamonds), independent of the CH_3_OH wt%.

The transition pressures for water-ice VI-ice VII at room temperature during compression and decompression cycles as a function of CH_3_OH wt% is summarized in Fig. 7. The crystallization pressure of water (filled circles) increases with increasing CH_3_OH wt% and saturates when the concentration of CH_3_OH is higher than 10 wt%. The transition pressure from ice VI to VII (filled squares), however, depends weakly on the concentration of CH_3_OH. When decreasing pressure, the ice VII in all the mixtures reversibly transformed back to ice VI at nearly the same pressures as during compression (open triangles), independent of the CH_3_OH wt%. The ice VI melted to water around 1 GPa (open diamonds) for all of the CH_3_OH wt% we studied, indicating a pronounced hysteresis for the water-ice VI phase transition in the mixtures.

**Figure 8:**
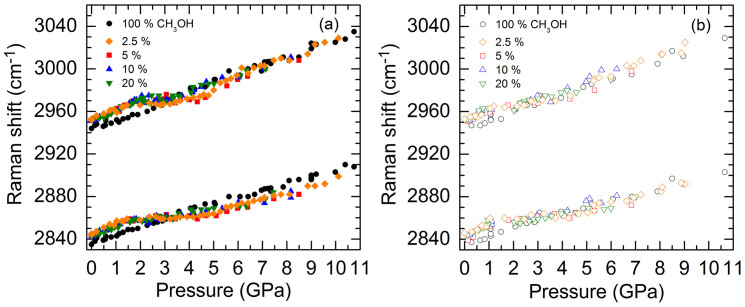
Raman shifts of the C-H stretching modes of CH_3_OH as a function of pressure and CH_3_OH wt % during (a) compression and (b) decompression cycles. The 100 wt%, pure CH_3_OH shows an approximately constant slope *dω*/*dP* in both compression and decompression cycles. The frequencies of both C-H modes for 2.5, 5, 10, and 20 wt% CH_3_OH are higher than those for pure CH_3_OH at ambient pressure, and initially increase linearly with a similar slope larger than those of pure CH_3_OH until the crystallization of water, in which the slopes all become very small. The frequencies then increase again at higher pressures with a slope similar to those of the pure CH_3_OH. The pressure induced frequency shifts in pure CH_3_OH are reversible over the pressure range studied, whereas the frequency shifts in all low CH_3_OH content mixtures are only reversible until around 1–2 GPa, where a hysteresis water-ice VI transition occurs and the small slopes of the C-H stretching modes of all low CH_3_OH content mixtures increase around 1 GPa.

To understand the effect of pressure on the molecular bonding and phase transition behaviors in H_2_O-CH_3_OH mixtures, we further compare the C-H stretching frequencies of CH_3_OH in pure methanol with those in H_2_O mixed with low (2.5, 5, 10 and 20 wt%) methanol content, as shown in Fig. 8. At ambient pressure, the C-H frequencies in all low methanol content mixtures are essentially the same and higher than the frequencies in pure methanol by ~9 cm^−1^ (Fig. 8a), in good agreement with previous studies^26^,^28^,^40^. Note that the C-O stretching frequencies (not shown) in all low methanol content mixtures (1017 cm^−1^) are the same and 14 cm^−1^ lower than the frequency in pure methanol (1031 cm^−1^)^26^,^28^,^40^,^41^. Upon compression, the C-H frequencies in low CH_3_OH content mixtures increase with similarly constant slopes *dω*/*dP* larger than the constant slopes of pure methanol until the crystallization of water at pressures that vary with the CH_3_OH wt%. The flattening of the C-H frequencies for 10 and 20 wt% CH_3_OH persisted until 3.6 GPa, lower than the pressure for 5 wt% (4.6 GPa) and 2.5 wt% CH_3_OH (5 GPa), after which all the frequencies increased with a constant slope similar to that of the pure methanol. Fig. 8b shows that the C-H frequencies reversibly shifted during decompression until approximately 1 GPa where the small slopes of all of the low methanol content mixtures started to increase. On the other hand, in the compression cycle, the decreasing rates of the O-H stretching frequencies of ice VII in all low methanol content mixtures were suppressed during a narrow pressure range, after which the frequencies red-shifted with nearly the same slope as that of pure H_2_O. Such evolution for C-H and O-H frequencies under pressure along with the observed pressure-induced variations in Raman intensities of these modes within the sample, and the hysteresis transition where the decompressed ice VI melted to water around 1 GPa as in the pure H_2_O (Fig. 7) suggest that the low content CH_3_OH molecules undergo a pressure-induced segregation from the H_2_O cage.

## Discussion

The C-H stretching frequencies of CH_3_OH are sensitive to local hydrogen-bonding configurations. In pure methanol, the CH_3_OH molecules form a series of chains and rings^20^,^28^,^42^, in which a CH_3_OH molecule accepts a hydrogen bond from a neighboring molecule and also donates a hydrogen to form another hydrogen bond. The C-H stretching frequencies increase when the CH_3_OH molecule serves as a proton acceptor (A) and the frequencies decrease when the CH_3_OH is a proton donor (D)^27^. On the other hand, in the H_2_O mixtures with low CH_3_OH content, a CH_3_OH molecule which is surrounded by an H_2_O cage accepts two hydrogen bonds from neighboring H_2_O molecules and donates a hydrogen atom to form a CH_3_OH-H_2_O hydrogen bond. Therefore, the transformation from the acceptor-donor (AD) configuration in pure CH_3_OH to the AAD configuration in low CH_3_OH content mixtures results in higher C-H frequencies in low CH_3_OH content mixtures at ambient pressure and a larger slope (*dω*/*dP*) under compression. When water crystallizes to ice VI, the H_2_O molecules form a tetragonal lattice arrangement that changes the local hydrogen-bonding configuration around the CH_3_OH molecule and suppresses the increase in the C-H stretching frequencies under pressure. Upon further compression, the structural transition from ice VI (tetragonal) to VII (cubic) does not significantly change the bonding around methanol molecules and thus the evolution of C-H frequencies with pressure remains nearly the same. The O-H stretching modes of ice VII soften with pressure and experience a reduction in the slopes during a narrow pressure range, which may be due to the pressure-driven perturbation of CH_3_OH molecules that move and segregate from the ice VII cages. This hypothesis is supported by the observation that in lower CH_3_OH content mixtures the reduction in the softening rates occurs at higher pressure regime. When large numbers of CH_3_OH molecules aggregates, the local hydrogen bonding configuration becomes similar to that in the pure CH_3_OH with an AD configuration. As a result, the C-H stretching frequencies increase and the O-H frequencies decrease with slopes similar to those of the pure CH_3_OH and ice VII, respectively.

## Conclusion

To summarize, we have used Raman spectroscopy to investigate the effect of pressure on the inter- and intra-molecular bonding in H_2_O-CH_3_OH mixtures. The presence of small amounts of CH_3_OH stabilizes the liquid water phase under pressure, but does not significantly affect the ice VI–VII phase transition, reducing the pressure range ice VI exists. Pressure evolution of the C-H stretching frequencies of CH_3_OH and the O-H stretching frequencies of H_2_O combined with the pressure-induced variations in the Raman intensities of these modes reveals changes in the hydrogen-bonding configuration in H_2_O-CH_3_OH mixtures and suggests a pressure-induced segregation of low content CH_3_OH from the ice VII. These findings could be general in other low temperature crystalline phases of ice as well as in other H_2_O-volatile mixtures, and are of importance for Earth and planetary sciences. For instance, within large icy satellites and potential icy super-Earths H_2_O may be in low temperature crystalline phases, e.g., ice II, VI, and VIII, and the segregation of volatiles from H_2_O ices will substantially reduce the thermal conductivity and sound velocity of H_2_O-volatile mixtures that would hinder the heat transfer through planetary interiors. The suppressed heat transfer would affect the thermal evolution and geodynamics processes, and prevent the sub-surface ocean from crystallizing within large icy bodies. In addition, the hysteresis of the water-ice VI transition may result in inhomogeneous mixing of crystalline ices and liquid water-volatile mixture near the boundary between icy layers and sub-surface ocean when the geothermal gradient within large icy bodies fluctuates due to the competition between the heat source from the core and from tidal dissipation within icy layers, and the heat sink through convection in the outer ice I layer. It is worth noting that such hysteresis cycle implies that re-melting occurs at higher temperatures than the initial crystallization temperature and thus would require a substantial amount of heat to occur^10^. We point out, however, that since a dramatic change in the slope *dω*/*dP* of the Raman frequency could be a signature of pressure-induced phase transition, we cannot exclude the possibility of additional, novel phase transitions in the H_2_O-CH_3_OH mixtures. Future X-ray and neutron diffraction experiments and computer simulations are necessary to provide complementary insights into the bonding behavior and phase relationships in the H_2_O-CH_3_OH mixtures under extreme pressures.

## Methods

Distilled H_2_O mixed with specific wt% of CH_3_OH was loaded, together with a ruby ball, into a symmetric diamond-anvil cell (DAC) with a culet size of 500 μm. The mixture itself served as a pressure transmitting medium. The pressure was determined by ruby fluorescence^43^ and the uncertainties of the pressure measurements were typically ~0.1 GPa. The compression and decompression rates of the samples were typically ~0.1 GPa s^−1^. After the pressure within the DAC equilibrated and reached a stable value, the high pressure Raman spectra of H_2_O-CH_3_OH mixtures were measured at room temperature using a Raman microscope (Horiba Jobin Yvon) that employs a CW 514.5 nm Ar-ion laser to excite the mixture within the DAC and probes the Raman scattering and vibrational frequency shifts with a spectral resolution of ~2 cm^−1^.

## Author Contributions

W.P.H. conceived and designed research. W.P.H. and Y.H.C. conducted the experiments and analyzed the data. W.P.H. wrote the manuscript. Both authors reviewed the manuscript.
